# Phosphate-Induced Reaction to Prepare Coal-Based P-Doped Hard Carbon with a Hierarchical Porous Structure for Improved Sodium-Ion Storage

**DOI:** 10.3390/molecules28134921

**Published:** 2023-06-22

**Authors:** Limin Deng, Yakun Tang, Jingmei Liu, Yue Zhang, Wenjun Song, Yuandong Li, Lang Liu

**Affiliations:** State Key Laboratory of Chemistry and Utilization of Carbon Based Energy Resources, College of Chemistry, Xinjiang University, Urumqi 830017, China; 107552100823@stu.xju.edu.cn (L.D.); yktang@xju.edu.cn (Y.T.); liujm@xju.edu.cn (J.L.); song@stu.xju.edu.cn (W.S.); lyd@stu.xju.edu.cn (Y.L.)

**Keywords:** sodium-ion batteries, coal-based hard carbon, phosphorus-dopes, carbon dissolution reaction, plateau capacity

## Abstract

The use of coal as a precursor for producing hard carbon is favored due to its abundance, low cost, and high carbon yield. To further optimize the sodium storage performance of hard carbon, the introduction of heteroatoms has been shown to be an effective approach. However, the inert structure in coal limits the development of heteroatom-doped coal-based hard carbon. Herein, coal-based P-doped hard carbon was synthesized using Ca_3_(PO_4_)_2_ to achieve homogeneous phosphorus doping and inhibit carbon microcrystal development during high-temperature carbonization. This involved a carbon dissolution reaction where Ca_3_(PO_4_)_2_ reacted with SiO_2_ and carbon in coal to form phosphorus and CO. The resulting hierarchical porous structure allowed for rapid diffusion of Na^+^ and resulted in a high reversible capacity of 200 mAh g^−1^ when used as an anode material for Na^+^ storage. Compared to unpretreated coal-based hard carbon, the P-doped hard carbon displayed a larger initial coulombic efficiency (64%) and proportion of plateau capacity (47%), whereas the unpretreated carbon only exhibited an initial coulombic efficiency of 43.1% and a proportion of plateau capacity of 29.8%. This work provides a green, scalable approach for effective microcrystalline regulation of hard carbon from low-cost and highly aromatic precursors.

## 1. Introduction

The major obstacle to boosting sodium-ion batteries’ (SIBs) industrialization lies in the lack of suitable anodes with excellent performance, low cost, and easy availability [1]. Among multifarious anode materials, hard carbons have been regarded as the most likely to be the first to achieve industrialization among many candidates because of their low sodium storage potential, moderate sodium storage capacity (higher than titanium-based materials and lower than alloy materials), small volume deformation after sodium insertion, and good cycle performance [2,3]. In addition, their short-range carbon microcrystalline and enlarged interlayer spacing ensure that more Na^+^ is inserted into the carbon lattice [4]. At present, various carbon precursors have been used in the synthesis of hard carbon, including waste biomass [5,6], organic polymers [7,8], coal [9,10,11], etc. However, the low carbon yield of waste biomass and expensive organic polymers hinder their practical application. Coal is recognized as a low-cost natural carbon source with high carbon conversion, abundant reserves, and wide distribution, making it an ideal hard carbon precursor [9,10,11,12,13,14]. However, as a thermoplastic carbon source, coal undergoes melting and rearrangement of organic polymers during pyrolysis, which facilitates the formation of graphite-like domains, which is unfavorable for Na^+^ storage [13]. Compared with anthracite, subbituminous coal has the advantages of low price, large reserves, and wide distribution. Moreover, subbituminous coal has a relatively high reactivity, which is more conducive to modulating carbon microcrystalline [15]. Recently, hard carbon anodes have been prepared by direct pyrolysis of anthracite [9] and bituminous coal [14], while their sodium storage performance is not satisfactory, which can be attributed to their highly graphitic structure and narrow interlayer spacing induced by high-temperature carbonization processes [16], resulting in the limitation of Na^+^ inserted into the graphite layers. Therefore, the key to preparing high-performance coal-based hard carbon anodes lies in orienting the microcrystalline structure during thermal conversion to achieve a short-range and loose microcrystalline structure [10,11].

Doping heteroatoms (such as nitrogen [17,18], fluorine [19], boron [7], sulfur [20,21], and phosphorus [5,6,22,23]) can effectively improve the Na-storage performance of carbon anodes, which could not only change the microstructure of carbon but also enlarge the interlayer spacing. Compared with other forms of heteroatom doping, phosphorus doping can achieve the enhancement of both plateau capacity and slope capacity, because it can enlarge interlayer spacing to facilitate adsorbing/inserting more Na^+^ [24,25] and increase defect sites to achieve a higher adsorption amount of Na^+^ [26]. Wu et al. [5] synthesized a flexible P-doped carbon cloth utilizing red phosphorus as the source of phosphorus, demonstrating that P-doping can ameliorate defects and oxygen functional groups, thereby augmenting sodium storage capacity and enhancing cycle stability. Dong et al. [22] synthesized P-doped nanofiber hard carbon through electrospinning, demonstrating that the introduction of phosphorus can effectively enhance interlayer spacing and pore structure, thereby facilitating rapid and efficient electron transport. Wang et al. [27] synthesize P-doped hollow carbon nanorods by the direct mechanical ball milling method to regulate the structure to improve its electrochemical performance. Although great progress has been made to provide a reversible capacity and provide new insights into designing the P-doped carbon anodes, these works used complex synthesis methods or flammable red phosphorus as phosphorus precursors to prepare P-doped carbon, which is difficult to achieve on a large scale. Therefore, it is necessary to find a simple, green, and efficient method to synthesize coal-based hard carbon with homogeneous P-doping for achieving microcrystalline regulation and larger interlayer distance.

Herein, subbituminous coal-based P-doped hard carbon was synthesized using Ca_3_(PO_4_)_2_ as a dopant through a carbon dissolution reaction. The Ca_3_(PO_4_)_2_ reacts with SiO_2_ and carbon in coal to form phosphorus and CO, which not only can obtain homogeneous phosphorus doping and a unique hierarchical porous structure but also inhibit the long-range development of carbon microcrystals during the high-temperature carbonization process, enabling this hard carbon with short microcrystals and large interlayer distance. The obtained hard carbon could deliver a reversible capacity of 200 mAh g^−1^ with a higher ICE. The electrochemical analysis shows that the low-voltage plateau capacity increases from 64 mAh g^−1^ to 89 mAh g^−1^ due to the enlargement of graphite layer spacing caused by continuous electrochemical activation, which is of great significance for achieving high-energy density SIBs.

## 2. Results

### 2.1. Structural Characterization

**Figure 1:**
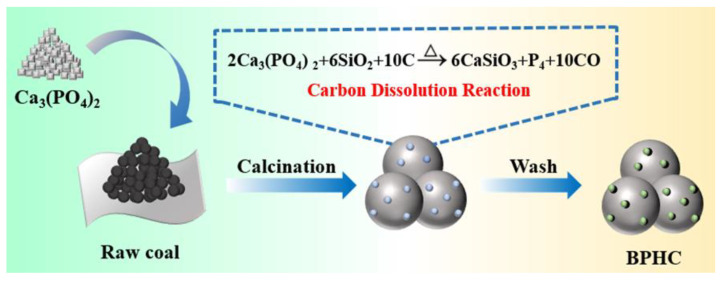
Schematic illustration of the synthesis procedure of BPHC.

The simple schematic illustration of the synthesis procedure of BPHC is shown in Figure 1. The carbon dissolution reaction equation is as follows:

(1) $$2{Ca}_{3}{{(PO}_{4})}_{2}+6{SiO}_{2}+10C\overset{\Delta }{\to }6{CaSiO}_{3}+P_{4}+10CO$$

**Figure 2:**
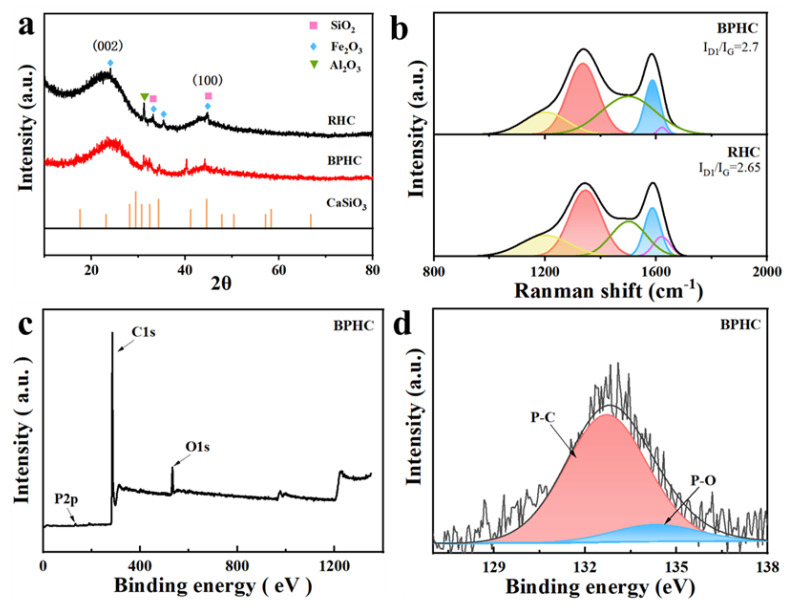
XRD patterns (**a**) and fitted Raman patterns (**b**) of RHC and BPHC. The full survey (**c**) and high-resolution (**d**) XPS spectra of BPHC. The black line and the red line are the original data; the blue line and pink line are the fitted data; and the gray line is the background.

The crystal characteristics of the RHC and BPHC samples were revealed by XRD (Figure 2a). Two obvious broad diffraction peaks located around 22° and 43° can be detected, which can be assigned to the (002) plane and the (100) plane of the typical characteristics of amorphous carbon [28,29]. The impurities of SiO_2_, Fe_2_O_3,_ and Al_2_O_3_ are all still present in the unpretreated RHC sample, while the impurities of Fe_2_O_3_ in BPHC are removed after the acid washing process; in addition, CaSiO_3_ can also be detected in the BPHC sample, indicating the occurrence of a carbon dissolution reaction based on Equation (1) and implying the formation of phosphorus and CO. The interlayer spacing of RHC and BPHC was calculated based on the (002) peak position by the Bragg equation (Equation (2)):

(2) $$d_{002}(nm)=\frac{n\lambda }{2sin\theta }$$

The transverse size La and stacking height Lc of carbon microcrystals are also calculated based on the fitted XRD model (Figure S1a) by the Debye–Schell equation (Equation (3)) [30]:

(3) $$L(nm)=\frac{k\lambda }{\beta cos\theta }$$

The calculated results are shown in Table S3. The (002) peak shifts from 23.95° to 23.82°, suggesting a more amorphous carbon microstructure with larger interlayer spaces of BPHC. The calculated lattice spaces of (002) of RHC and BPHC are 3.71 and 3.73 Å, respectively, which are all larger than that of graphite (3.35 Å). The values of Lc and La of BPHC are smaller than those of RHC, demonstrating that this phosphate-induced carbon dissolution reaction can effectively hinder the orderly rearrangement of carbon layers and reduce the crystallite size of coal-derived carbon, and the larger average carbon layer distance is in favor of enhancing the diffusion and storage of Na^+^ to carbon microcrystalline [31].

Furthermore, the Raman spectra of RHC and BPHC can be fitted into five sub-peaks located at 1200, 1350, 1500, 1580, and 1620 cm^−1^. The corresponding fitted information and results are shown in Figure 2b and Table S4 [32]. The integrated area of the I_D1_/I_G_ subpeak can be used to indicate the degree of disorder in the carbon structure [33,34]. The I_D1_/I_G_ values for RHC and BPHC are 2.65 and 2.7, respectively. The larger I_D1_/I_G_ value of BPHC demonstrates a more disordered carbon structure than that of RHC, which is also consistent with the XRD results. The result of the Raman spectra suggests that the carbon dissolution reaction could achieve P-doping into the carbon framework and prevent the evolution of the graphite-like microcrystalline structure during high-temperature carbonization, leading to a decrease in the degree of graphitization and an increase in defects in BPHC. XPS was used to determine the surface chemical elements and binding states of BPHC (Figure 2c). The XPS spectrum of BPHC exhibits three peaks, corresponding to O1s, P1s, and C1s. The fitted phosphorus content of BPHC was at 0.41%. The P2p spectrum can be deconvoluted into two sub-peaks that correspond to the P-C bond (132.7 eV) and the P-O bond (134.3 eV), and the fitted proportion of the P-C bond is up to 85%, indicating the successful introduction of phosphorus atoms in hard carbon, and the phosphorus atoms are mainly linked to carbon atoms. The high proportion of the P-C bond is more favorable to enlarging the carbon layer distance of BPHC and facilitates Na^+^ transfer [35,36]. The energy dispersive spectroscopy (EDS) mapping of BPHC is displayed in Figure S2, revealing the uniform dispersion of C, O, P, Ca, and Si elements on the surface of BPHC, again confirming the formation of CaSiO_3_ and successful phosphorus doping by the carbon dissolution reaction. In addition, EDS quantification indicates that the content of P in BPHC is 3.89 wt% (Figure S3).

**Figure 3:**
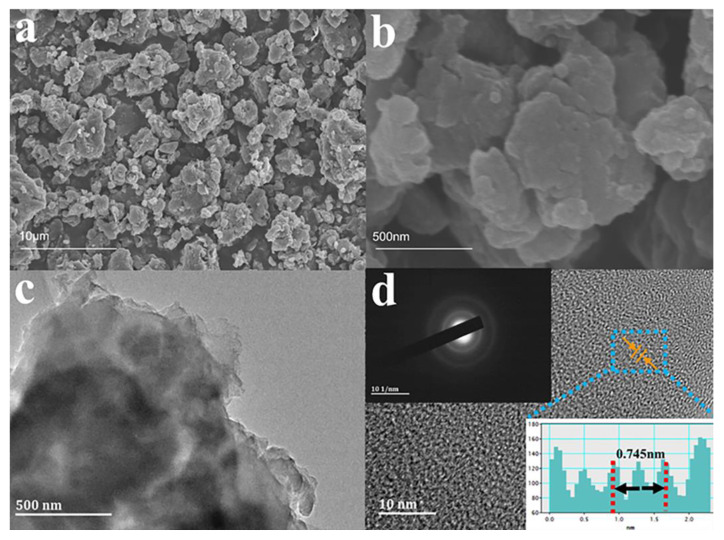
SEM images at different magnifications (**a**,**b**), TEM image (**c**), HRTEM image with corresponding intensity profile for the line scan across the lattice fringes (**d**), and SAED (inserted in (**d**)) image of BPHC.

SEM and TEM measurements were performed to investigate the structural changes of the obtained samples. As shown in Figure 3a,b and Figure S4a,b, after pre-treatment by this carbon dissolution reaction, the particle size of the material is obviously reduced, and the surface is rich in the pore structure. Smaller particle sizes and rich pore structures are more conducive to the rapid transmission of Na^+^ [37,38,39]. TEM analysis confirms that BPHC possesses a porous structure from the inside to the outside (Figure 3c), which could be attributed to the homogeneous carbon dissolution reaction. The HRTEM image of Figure 3d shows BPHC has wide layer spacing and closed pores formed by curled graphite layers, which belong to a typical pseudo-graphitic structure. The interlayer spacing of the randomly stacked curly graphite layer is measured at about 3.725 Å, which is consistent with the results of XRD. The pseudo-graphitic structure promotes interlayer intercalation and pore-filling of sodium ions to increase its plateau capacity. The dispersed diffraction rings shown in the SAED image also confirm that BPHC is a typical amorphous carbon material (inserted in Figure 3d).

**Figure 4:**
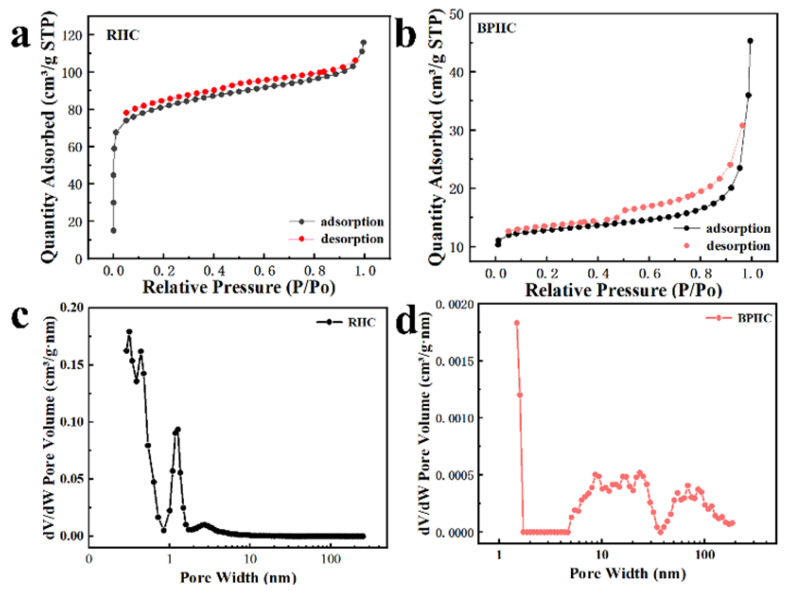
The N_2_ adsorption/desorption isotherms (**a**,**b**) and their corresponding pore-size distributions (**c**,**d**) of RHC and BPHC.

The N_2_ adsorption/desorption measurements were performed to analyze the specific surface area and porosity of the two samples [40]. As shown in Figure 4a,b, RHC and BPHC show significantly different N_2_ adsorption/desorption isotherms in Figure 4a,b. RHC exhibits type I isotherms, and the adsorption capacity rises rapidly at lower relative pressure, which belongs to microporous filling, indicating that it has a rich microporous structure. BPHC exhibits type IV isotherms, showing a hysteresis loop at a high P/P_0_ range of adsorption/desorption isotherms, indicating the existence of mesopores in the sample. The BET-specific surface areas of the RHC and BPHC samples are 307 m^2^ g^−1^ and 49.6 m^2^ g^−1^, respectively. The higher specific surface area of RHC could be attributed to the spill of a large number of light components from subbituminous coal during heat treatment, causing pore structures in the hard carbon. Such a high specific surface area of RHC will bring lots of surface defects, resulting in a low initial coulombic efficiency (ICE). Figure 4c,d shows the pore size distribution based on the DFT model fits for RHC and BPHC. RHC is mainly composed of micropores and a few mesopores, whereas BPHC has a continuous pore size distribution from micropores to meso- and macropores. The micropores could accept electrons and electron-donating species, the mesopores provide diffusion channels for Na^+^ to migrate toward the micropores, and the macropores work as buffer reservoirs for electrolyte ions [41]. Therefore, combining micropores with meso/macropores to construct hierarchical porous carbon materials (BPHC) can achieve excellent electrochemical stability and high-rate performance.

### 2.2. Electrochemical Properties

**Figure 5:**
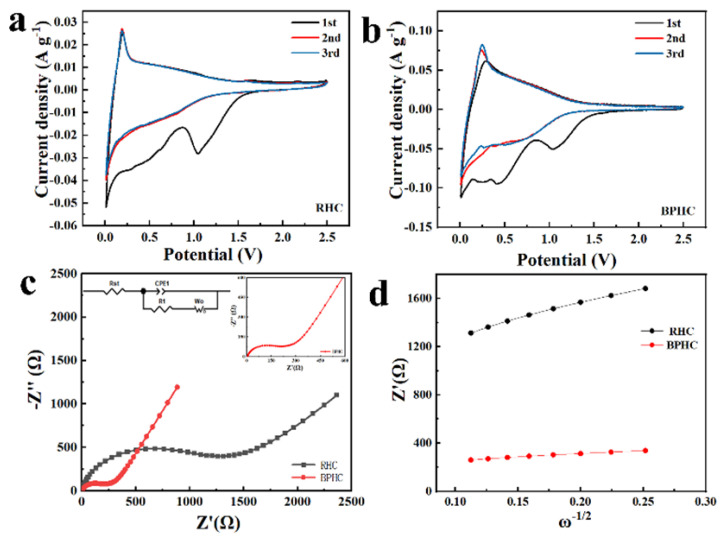
CV curves (**a**,**b**), Nyquist plots (**c**), and the relationship between Z′ and ω^−1/2^ in the low-frequency region (**d**) of RHC and BPHC.

The cyclic voltammogram (CV) curves of RHC and BPHC in the potential range of 0.01 V–2.5 V (vs. Na/Na^+^) at a scan rate of 0.1 mV/s are shown in Figure 5a,b. The curves show the typical characteristics of carbon materials. Apart from the initial curve, the second and third CV curves have similar shapes and overlapped well, suggesting that the electrodes have good electrochemical reversibility. The irreversible peaks located at 0.5 V and 1.0 V during the initial cycles disappear in subsequent cycles due to electrolyte decomposition and the formation of solid–electrolyte interfaces (SEIs) film [37,42]. The oxidation/reduction peak at around 0.1 V corresponds to the insertion/desertion of Na^+^ between the graphite layers. It is obvious that the oxidation/reduction peak of BPHC is sharper than that of RHC, which is due to the fact that P-doping facilitates the formation of blistering in its structure, which is conducive to the adsorption/insertion of more Na^+^ and the widening of interlayer spacing to promote Na^+^ transfer [36]. Figure 5c shows the EIS of RHC and BPHC electrodes with the frequency ranging from 0.01 Hz to 1.0 MHz, and the EIS curves were fitted based on an equivalent electric circuit inserted in Figure 5c.

The Nyquist plots include curved curves in the high-frequency range and sloping straight lines in the low-frequency range, corresponding to charge transfer resistance (Rct) and Warburg impedance, respectively. The relationship between Z_re_ and ω^−1/2^ is shown in Figure 5d, the Weber coefficient (σ) can be calculated based on Equation (4), and the Na^+^ diffusion coefficient (D_Na+_) can be calculated based on Equation (5) [43].

(4) $$Z_{re}=R_{s}+R_{ct}+\sigma \omega^{-1/2}$$

(5) $$D_{{Na}^{+}}=\frac{R^{2}T^{2}}{2A^{2}n^{4}F^{4}C_{Na}^{2}\sigma^{2}}$$

The results of the calculations are presented in Table S5. Compared to the RHC electrode, the smaller R_ct_ and larger Na^+^ diffusion coefficient (*D*_Na+_) of the BPHC electrode imply that there is faster transport of both electrons and Na^+^.

**Figure 6:**
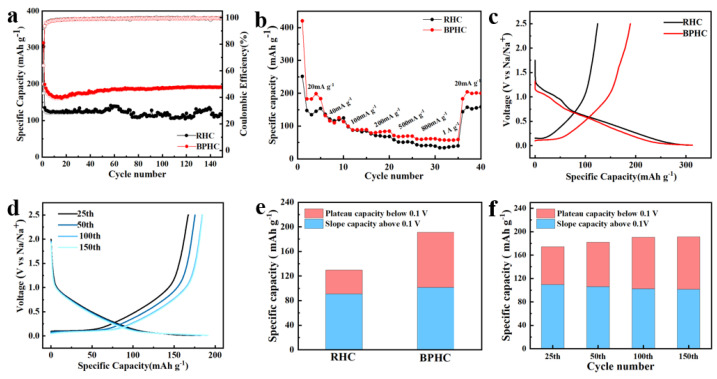
Rate performance from 20 to 1000 mA g^−1^ (**a**). Cycle performance at 100 mA g^−1^ (**b**). The initial discharge curves at 100 mA g^−1^ (**c**). The charge/discharge curves in different cycles at 100 mA g^−1^ of BPHC (**d**). Plateau region capacity and sloping region capacity comparison after 150 cycles of RHC and BPHC (**e**), and plateau region capacity and sloping region capacity comparison at different cycles of BPHC (**f**).

The sodium storage properties of RHC and BPHC were evaluated in sodium half-cells between the voltage range of 0.01–2.5 V. Figure 6a shows the rate performance of RHC and BPHC at different current densities from 20 to 1000 mA g^−1^. Among them, BPHC shows the optimal rate performance with reversible capacities of 183.6, 111.1, 97.6, 86.8, 71.4, 62.1, and 58.2 mAh g^−1^ at 20, 40, 100, 200, 500, 800, and 1000 mA g^−1^, respectively. When the current density returns to 20 mA g^−1^, the BPHC still has a high capacity of 200 mAh g^−1^. The RHC exhibits poor rate performance, achieving only 34 mAh g^−1^ at 1000 mA g^−1^, while the BPHC displays better rate performance (58.2 mAh g^−1^) due to the hierarchical porous structure that provides channels for electrolytes, Na^+^, and fast electron transfer, leading to high specific capacity and excellent electric conductivity. The cycling charge/discharge performance of the RHC and BPHC electrodes at a current density of 100 mA g^−1^ is shown in Figure 6b. BPHC shows a significant decrease in initial capacity, probably because Na^+^ storage occurs mainly in the near-surface region of the active material, and therefore, BPHC as a micron-sized hard carbon material requires more cycles to form a stable and complete SEI layer [44,45,46]. Therefore, although the material has uniform phosphorus doping, an electrochemical activation process is still required. Subsequently, with the increase in the cycle number, the capacity gradually increased and finally stabilized, which can be attributed to the electrochemical activation process of the electrode. Notably, a high reversible capacity of up to 190 mAh g^−1^ can be achieved even after 150 cycles, while the RHC electrode only discharges a capacity of 130 mAh g^−1^. The initial charge/discharge curves of two samples at 100 mA g^−1^ are shown in Figure 6c, from which the ICE of RHC and BPHC can be calculated to be 43.1 % and 64 %, respectively. The higher ICE of BPHC can be associated with its smaller specific surface area (49.6 m^2^ g^−1^), resulting in fewer surface defects. Figure 6d shows the charging and discharging curves of BPHC at different numbers of cycles, the plateau region capacity increases with the increasing number of cycles, and the polarization effect gradually decreases, demonstrating the electrochemical activation process during cycling, and the increased capacity may be derived from Na^+^ insertion/desertion in the low-voltage region.

Figure 6e shows a comparison of the plateau region capacity and sloping region capacity at the 150th lap, with BPHC (47%) demonstrating a higher plateau region capacity than RHC (29.8%). The increased capacity of the BPHC plateau is due to homogeneous P-doping in the carbon dissolution reaction, resulting in increased interlayer spacing and more favorable insertion of Na^+^ into pseudo-graphite domains. The improved electrochemical performance of BPHC is mainly attributed to the porous hierarchical structure formed by the carbon dissolution reaction offering a fast transport channel for Na^+^. In addition, this reaction can promote homogeneous P-doping, further expanding graphite layer spacing and providing a suitable interlayer space for Na^+^ intercalation. A comparison between the plateau region capacity and sloping region capacity during different cycles of the BPHC electrode is shown in Figure 6f, indicating that the increased capacity is primarily attributed to the plateau capacity. The plateau capacity increased from 64 mAh g^−1^ at the 25th cycle to 89 mAh g^−1^ at the 150th cycle. Notably, the similar plateau capacity of the 100th and 150th circles shows that it does not increase over time, demonstrating acceptable structural tolerance and reversibility.

**Figure 7:**
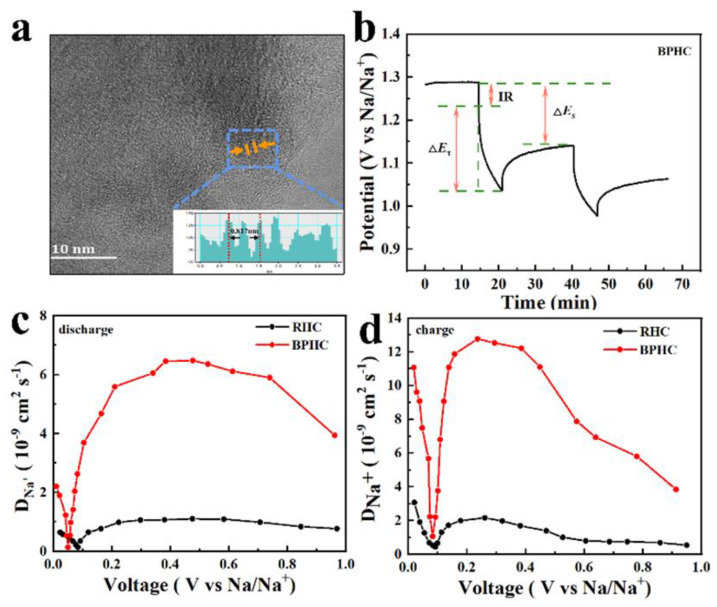
HRTEM image with the corresponding intensity profile for the line scan across the lattice fringes of BPHC at 100 mAh g^−1^ after 150 cycles (**a**); scheme for a single step of the GITT experiment (**b**); and the calculated Na^+^ chemical diffusion coefficients of the RHC and BPHC electrodes during the discharge (**c**) and charge (**d**) process.

The HRTEM image of the BPHC sample after 150 cycles at 100 mAh g^−1^ is also provided. As shown in Figure 7a, the sample still presents a typical curly microstructure with larger carbon clusters after cycling. Importantly, the graphite layer spacing expands to 0.408 nm, indicating that the structural changes caused by the (de)intercalation of Na are irreversible [47]. The enlarged layer spacing is associated with full activation of the micron-sized bulk material through a continuous electrical activation process, resulting in P-doping acting both on the surface and in the interior of the material, contributing to the formation of more blistering in the structure, thereby widening the spacing of the graphite layers and promoting more Na^+^ in/de-insertion into the overall material. This result is consistent with the gradual increase in plateau capacity as the number of cycles increases. The galvanostatic intermittent titration technique (GITT) was also used to understand the kinetics of Na^+^ storage in the RHC and BPHC electrodes with a pulse current at 20 mA g^−1^ for 10 min between a rest interval of 30 min. Based on Fick’s second law, the *D*_Na_^+^ could be calculated through Equation (6) [48,49,50]:

(6) $$D_{{Na}^{+}}=\frac{4}{\pi \tau }{\left(\frac{m_{c}V_{c}}{M_{c}S}\right)}^{2}({\frac{\Delta E_{s}}{\Delta E_{t}})}^{2}$$

ΔE_s_ is the difference between the open circuit potentials and ΔE_t_ is the voltage change during the current pulse application. The values of ΔE_s_ and ΔE_t_ depend on each current step shown in Figure 7b. The *D*_Na_^+^ of the RHC and BPHC electrodes during discharge and charge are presented in Figure 7c,d, respectively. Both two GITT curves show the same tendency; in the discharge process, the *D*_Na_^+^ of BPHC increases slowly to 6.6 × 10^−9^ cm^2^ s^−1^ in the voltage range from 1.0 to 0.5 V, which corresponds to the adsorption process of Na^+^ on the active site and defect site of the material surface. *D*_Na_^+^ decreased slowly in the voltage range of 0.5–0.2 V, indicating that the surface adsorption sites begin to decrease. The *D*_Na_^+^ of BPHC dropped sharply to 0.25 × 10^−9^ cm^2^ s^−1^ in the voltage range of 0.2–0.1 V, because Na^+^ could only intercalate into graphite layers after the active sites and defect site on the surface are completely occupied by Na^+^, and this process need to overcome larger energy barriers to insert Na^+^ into graphite layers [51]. After overcoming this energy barrier, the insertion of Na^+^ becomes easier, thus the *D*_Na_^+^ shows an increasing trend after 0.1 V. The overall trend of the charging process is opposite to that of the discharge process, which proves Na^+^ insertion/desertion in the low voltage (<0.1 V). In short, the BPHC electrode has higher *D*_Na_^+^ than that of the RHC electrode during the charging and discharging process. Compared with the reported literature [37,52], the *D*_Na_^+^ of the BPHC electrode is also relatively high. The high *D*_Na_^+^ can be attributed to the hierarchical porous structure and the large graphite layer spacing, which facilitates electrolyte penetration and the adsorption and insertion of Na^+^.

## 3. Materials and Methods

### 3.1. Materials Preparation

Subbituminous coal from Kucha in Xinjiang, China, was used as the precursor for hard carbon preparation. The proximate, ultimate, and ash composition analyses of subbituminous coal are shown in Tables S1 and S2. The content of volatile components in subbituminous coal is 35.08 wt%, which can be attributed to the presence of small organic molecules in coal, and the content of ash (inorganic impurities) is 3.76 wt%, which consists mainly of Fe_2_O_3_, SiO_2,_ and Al_2_O_3,_ etc. Typically, 1 g raw coal was ground to powder of about 200 mesh size, and then ball milled together with 0.2 g Ca_3_(PO_4_)_2_ for 3 h. The pretreated coal was annealed at 350 °C for 3 h and then heated at 1000 °C for 4 h under an Ar atmosphere. Finally, the carbonized mixtures were washed with HCl (6 M) to remove impurities and repeatedly washed with deionized water, which was termed as BPHC. For comparison, a control experiment was also performed. The hard carbon denoted as RHC was prepared by direct pyrolysis of subbituminous coal with the same pyrolysis process of BPHC.

### 3.2. Characterization Instruments

The structures of two samples were analyzed by X-ray diffraction (XRD, Bruker D8, Bruker Corporation, Mannheim, Germany) and a Raman spectroscope (Bruker SENTER, Bruker Corporation, Mannheim, Germany). Their structures were characterized by a field emission scanning electron microscope (SEM, Hitachi, Tokyo, Japan) and transmission electron microscopy (TEM, FEITecnaiF30, FEI, San Juan Capistrano, CA, USA). EDS mapping was used to analyze the elements. The lattice characteristics of the samples were studied by a high-resolution transmission electron microscope (HRTEM, JEM-2100F, Hitachi, Japan), and the crystal morphology of the samples was characterized by selective diffraction. The element composition, bonding type, and other chemical structures of the sample were analyzed by using X-ray photoelectron spectroscopy (XPS, ESCALAB 250Xi, Thermo Fisher Scientific, Waltham, MA, USA). Nitrogen adsorption/desorption isotherms were determined by nitrogen physisorption (ASAP 2460, Micromeritics, Norcross, GA, USA). The specific surface areas were estimated according to the Brunauer-Emmet–Teller (BET) method, and the pore size distribution was calculated by the density functional theory (dFt) method.

### 3.3. Electrochemical Characterization

All the electrochemical tests were conducted in button cells (LIR2025) assembled in an argon-filled glove box (O_2_ < 0.01 ppm, H_2_O < 0.01 ppm). The working electrode was prepared by spreading the mixed slurry of the active material, acetylene black as the conductive carbon, and polyvinylidene fluoride (PVDF) as a binder in N-methyl pyrrolidine (NMP) with a weight ratio of 8:1:1 onto a copper foil current collector and then dried at 110 °C in a vacuum oven for 12 h. The electrolyte is 1.0 M NaClO4 in EC:PC (1:1 vol% with 5.0% FEC). Cyclic voltammetry (CV) and electrochemical impedance spectroscopy (EIS) were carried out on an electrochemical workstation (CHI660E, CHI Company, Shanghai, China). The voltage range of CV is 0.01–2.5 V, and the scan rate is 0.1 mV s^−1^. EIS was carried out at a frequency ranging from 1.0 MHz to 0.01 Hz, and the amplitude was 10.0 mV.

## 4. Conclusions

In conclusion, we propose a mild and scalable approach to synthesize P-doped coal-based hard carbon with a hierarchical porous structure through the carbon dissolution reaction. The improved electrochemical performance of BPHC is mainly attributed to the carbon dissolution reaction of Ca_3_(PO_4_)_2_, which provides an admirable transport channel for Na^+^, inhibits the rearrangement of carbon microcrystals during coalification, and contributes to the formation of homogeneous P-doping. As a result, the BPHC electrode could deliver a high reversible capacity of 200 mAh g^−1^ with a higher ICE of up to 64%. Interestingly, during cycling, the plateau capacity increased from 64 mAh g^−1^ to 89 mAh g^−1^ due to the enlarged interlayer spacing caused by the continuous activation of P-doped coal-based hard carbon materials during the cycle. We believe that this mild pretreatment method will provide a new perspective to optimize the performance of coal-based hard carbons.

## Data Availability

Data will be made available upon request.

## Supplementary Materials

The following supporting information can be downloaded at: https://www.mdpi.com/article/10.3390/molecules28134921/s1, Table S1: The proximate and ultimate analyses of subbituminous coal. Table S2: The ash composition analyses (wt%) of subbituminous coal. Table S3: The XRD (002) peak fitting analysis for RHC and BPHC. Table S4: Deconvoluted band assignments of Raman spectra. Table S5: Data calculated by EIS of RHC and BPHC. Figure S1: Fitted XRD patterns and Raman spectra of RHC and BPHC. Figure S2: SEM and EDS elemental mappings of BPHC; SEM image of BPHC (a); elemental mappings for (b) C, (c) O, (d) Ca, (e) P and (f) Si of BPHC based on (a). Figure S3: EDS quantification of C, O, P, Ca and Si elements for BPHC. Figure S4: SEM images at different magnifications of RHC (a,b).

## Sample Availability

Samples are available from the authors upon request.

## References

[1] Pu X., Wang H., Zhao D., Yang H., Ai X., Cao S., Chen Z., Cao Y. (2019). Recent Progress in Rechargeable Sodium-Ion Batteries: Toward High-Power Applications. Small.

[2] Hou H., Qiu X., Wei W., Zhang Y., Ji X. (2017). Carbon anode materials for advanced sodium-ion batteries. Adv. Energy Mater..

[3] Chen X., Liu C., Fang Y., Ai X., Zhong F., Yang H., Cao Y. (2022). Understanding of the sodium storage mechanism in hard carbon anodes. Carbon Energy.

[4] Cheng D., Zhou X., Hu H., Li Z., Chen J., Miao L., Ye X., Zhang H. (2021). Electrochemical storage mechanism of sodium in carbon materials: A study from soft carbon to hard carbon. Carbon.

[5] Lu H., Zhang X., Wan F., Liu D., Fan C., Xu H., Wang G., Wu X. (2017). Flexible P-Doped Carbon Cloth: Vacuum-Sealed Preparation and Enhanced Na-Storage Properties as Binder-Free Anode for Sodium Ion Batteries. ACS Appl. Mater. Interfaces.

[6] Tian W., Wang L., Huo K., He X. (2019). Red phosphorus filled biomass carbon as high-capacity and long-life anode for sodium-ion batteries. J. Power Sources.

[7] Wu D., Sun F., Qu Z., Wang H., Lou Z., Wu B., Zhao G. (2022). Multi-scale structure optimization of boron-doped hard carbon nanospheres boosting the plateau capacity for high performance sodium ion batteries. J. Mater. Chem. A.

[8] Zhang G., Zhang L., Ren Q., Yan L., Zhang F., Lv W., Shi Z. (2021). Tailoring a Phenolic Resin Precursor by Facile Pre-oxidation Tactics to Realize a High-Initial-Coulombic-Efficiency Hard Carbon Anode for Sodium-Ion Batteries. ACS Appl. Mater. Interfaces.

[9] Li Y., Hu Y., Qi X., Rong X., Li H., Huang X., Chen L. (2016). Advanced sodium-ion batteries using superior low cost pyrolyzed anthracite anode: Towards practical applications. Energy Storage Mater..

[10] Wang K., Sun F., Wang H., Wu D., Chao Y., Gao J., Zhao G. (2022). Altering Thermal Transformation Pathway to Create Closed Pores in Coal-Derived Hard Carbon and Boosting of Na+ Plateau Storage for High-Performance Sodium-Ion Battery and Sodium-Ion Capacitor. Adv. Funct. Mater..

[11] Lou Z., Wang H., Wu D., Sun F., Gao J., Lai X., Zhao G. (2022). Microcrystalline regulation of bituminous coal derived hard carbon by pre-oxidation strategy for improved sodium-ion storage. Fuel.

[12] Zhao H., Zhao D., Ye J., Wang P., Chai M., Li Z. (2022). Directional Oxygen Functionalization by Defect in Different Metamorphic-Grade Coal-Derived Carbon Materials for Sodium Storage. Energy Environ. Sci..

[13] Chen H., Sun N., Zhu Q., Soomro R., Xu B. (2022). Microcrystalline Hybridization Enhanced Coal-Based Carbon Anode for Advanced Sodium-Ion Batteries. Adv. Sci..

[14] Lu H., Sun S., Xiao L., Qian J., Ai X., Yang H., Lu A., Cao Y. (2019). High-Capacity Hard Carbon Pyrolyzed from Subbituminous Coal as Anode for Sodium-Ion Batteries. ACS Appl. Energy Mater..

[15] Mathews J., Chaffee A. (2012). The molecular representations of coal—A review. Fuel.

[16] Sun F., Wang H., Qu Z., Wang K., Wang L., Gao J., Gao J., Liu S., Lu Y. (2021). Carboxyl-Dominant Oxygen Rich Carbon for Improved Sodium Ion Storage: Synergistic Enhancement of Adsorption and Intercalation Mechanisms. Adv. Energy Mater..

[17] Yan Z., Yang Q., Wang Q., Ma J. (2020). Nitrogen doped porous carbon as excellent dual anodes for Li- and Na-ion batteries. Chin. Chem. Lett..

[18] Zhu J., Chen C., Lu Y., Ge Y., Jiang H., Fu K., Zhang X. (2015). Nitrogen-doped carbon nanofibers derived from polyacrylonitrile for use as anode material in sodium-ion batteries. Carbon.

[19] Wang P., Qiao B., Du Y., Li Y., Zhou X., Dai Z., Bao J. (2015). Fluorine-Doped Carbon Particles Derived from Lotus Petioles as High-Performance Anode Materials for Sodium-Ion Batteries. J. Phys. Chem. C.

[20] Yang J., Zhou X., Wu D., Zhao X., Zhou Z. (2017). S-Doped N-Rich Carbon Nanosheets with Expanded Interlayer Distance as Anode Materials for Sodium-Ion Batteries. Adv. Mater..

[21] Hong Z., Zhen Y., Ruan Y., Kang M., Zhou K., Zhang J., Huang Z., Wei M. (2018). Rational Design and General Synthesis of S-Doped Hard Carbon with Tunable Doping Sites toward Excellent Na-Ion Storage Performance. Adv. Mater..

[22] Wu F., Dong R., Bai Y., Li Y., Chen G., Wang Z., Wu C. (2018). Phosphorus-Doped Hard Carbon Nanofibers Prepared by Electrospinning as an Anode in Sodium-Ion Batteries. ACS Appl. Mater. Interfaces.

[23] Song W., Tang Y., Liu J., Xiao S., Zhang Y., Gao Y., Yang C., Liu L. (2023). Mild pretreatment synthesis of coal-based phosphorus-doped hard carbon with extended plateau capacity as anodes for sodium-ion batteries. J. Alloys Compd..

[24] Hou H., Shao L., Zhang Y., Zou G., Chen J., Ji X. (2017). Large-Area Carbon Nanosheets Doped with Phosphorus: A High-Performance Anode Material for Sodium-Ion Batteries. Adv. Sci..

[25] Qiao Y., Han R., Pang Y., Lu Z., Zhao J., Cheng X., Zhang H., Yang Z., Yang S., Liu Y. (2019). 3D well-ordered porous phosphorus doped carbon as an anode for sodium storage: Structure design, experimental and computational insights. J. Mater. Chem. A.

[26] Thompson M., Xia Q., Hu Z., Zhao X. (2021). A review on biomass-derived hard carbon materials for sodium-ion batteries. Mater. Adv..

[27] Wang X., Hou M., Shi Z., Liu X., Mizota I., Lou H., Wang B., Hou X. (2021). Regulate Phosphorus Configuration in High P-Doped Hard Carbon as a Superanode for Sodium Storage. ACS Appl. Mater. Interfaces.

[28] Liu T., Luo R., Yoon S., Mochida I. (2010). Anode performance of boron-doped graphites prepared from shot and sponge cokes. J. Power Sources.

[29] Xing B., Guo H., Chen L., Chen Z., Zhang C., Huang G., Xie W., Yu J. (2015). Lignite-derived high surface area mesoporous activated carbons for electrochemical capacitors. Fuel Process. Technol..

[30] Shiraishi M., Inagaki M. (2003). Chapter 10-X-ray diffraction methods to study crystallite size and lattice constants of carbon materials. Carbon Alloys: Novel Concepts to Develop Carbon Science and Technology.

[31] Wen Y., He K., Zhu Y., Han F., Xu Y., Matsuda I., Ishii Y., Cumings J., Wang C. (2014). Expanded graphite as superior anode for sodium-ion batteries. Nat. Commun..

[32] Ferrari A., Robertson J. (2000). Interpretation of Raman spectra of disordered and amorphous carbon. Phys. Rev. B.

[33] Tsai P., Chung S., Lin S., Yamada A. (2015). Ab initio study of sodium intercalation into disordered carbon. J. Mater. Chem. A.

[34] Jeon J., Zhang L., Lutkenhaus J., Laskar D., Lemmon J., Choi D., Nandasiri M., Hashmi A., Xu J., Motkuri R. (2015). Cover Picture: Controlling Porosity in Lignin-Derived Nanoporous Carbon for Supercapacitor Applications. ChemSusChem.

[35] Yan J., Li H., Wang K., Jin Q., Lai C., Wang R., Jiang K. (2021). Ultrahigh Phosphorus Doping of Carbon for High-Rate Sodium Ion Batteries Anode. Adv. Energy Mater..

[36] Zhao H., Hu Z., Zhu Y., Ge L., Yuan Z. (2019). P-doped mesoporous carbons for high-efficiency electrocatalytic oxygen reduction. Chin. J. Catal..

[37] Alvin S., Chandra C., Kim J. (2020). Extended plateau capacity of phosphorus-doped hard carbon used as an anode in Na- and K-ion batteries. Chem. Eng. J..

[38] Xia J., Lu A., Yu X., Li W. (2021). Rational Design of a Trifunctional Binder for Hard Carbon Anodes Showing High Initial Coulombic Efficiency and Superior Rate Capability for Sodium-Ion Batteries. Adv. Energy Mater..

[39] Li G., Guo S., Xiang B., Mei S., Zheng Y., Zhang X., Gao B., Chu P., Huo K. (2022). Recent advances and perspectives of microsized alloying-type porous anode materials in high-performance Li- and Na-ion batteries. Energy Mater..

[40] He Q., Ding L., Raheem A., Guo Q., Gong Y., Yu G. (2021). Kinetics comparison and insight into structure-performance correlation for leached biochar gasification. Chem. Eng. J..

[41] Zhang Y., Huang Y., Wang X., Guo Y., Jia D., Tang X. (2015). Improved electrochemical performance of lithium iron phosphate in situ coated with hierarchical porous nitrogen-doped graphene-like membrane. J. Power Sources.

[42] Fan L., Lu B. (2016). Reactive Oxygen-Doped 3D Interdigital Carbonaceous Materials for Li and Na Ion Batteries. Small.

[43] Zhu Y., Zhang R., Deng L., Yi T., Ye M., Yao J., Dai C. (2015). Lithium-Ion Insertion Kinetics of Na-Doped LiFePO_4_ as Cathode Materials for Lithium-Ion Batteries. Metall. Mater. Trans. E-Mater. Energy Syst..

[44] Zhao G., Zou G., Qiu X., Li S., Guo T., Hou H., Ji X. (2017). Rose-like N-doped Porous Carbon for Advanced Sodium Storage. Electrochim. Acta.

[45] Wu Y., Wei Y., Wang J., Jiang K., Fan S. (2013). Conformal Fe_3_O_4_ sheath on aligned carbon nanotube scaffolds as high-performance anodes for lithium ion batteries. Nano Lett..

[46] Selvamani V., Gopi S., Rajagopal V., Kathiresan M., Vembu S., Velayutham D., Gopukumar S. (2017). High rate performing in situ nitrogen enriched spherical carbon particles for Li/Na-ion cells. ACS Appl. Mater. Interfaces.

[47] Zhou Y., Xiao Z., Han D., Wang S., Chen J., Tang W., Yang M., Shao L., Shu C., Hua W. (2023). Inhibition of the P3–O3 phase transition via local symmetry tuning in P3-type layered cathodes for ultra-stable sodium storage. J. Mater. Chem. A.

[48] Yan Y., Yin Y., Guo Y., Wan L. (2014). A Sandwich-Like Hierarchically Porous Carbon/Graphene Composite as a High-Performance Anode Material for Sodium-Ion Batteries. Adv. Energy Mater..

[49] Li Y., Hu Y., Titirici M., Chen L., Huang X. (2016). Hard Carbon Microtubes Made from Renewable Cotton as High-Performance Anode Material for Sodium-Ion Batteries. Adv. Energy Mater..

[50] Zhang J., Wan J., Ou M., Liu S., Huang B., Xu J., Sun S., Xu Y., Lin Y., Fang C. (2023). Enhanced all-climate sodium-ion batteries performance in a low-defect and Na-enriched Prussian blue analogue cathode by nickel substitution. Energy Mater..

[51] Bommier C., Surta T., Dolgos M., Ji X. (2015). New Mechanistic Insights on Na-Ion Storage in Nongraphitizable Carbon. Nano Lett..

[52] Zhang Y., Li L., Xiang Y., Zou G., Hou H., Deng W., Ji X. (2020). High Sulfur-Doped Hard Carbon with Advanced Potassium Storage Capacity via a Molten Salt Method. ACS Appl. Mater. Interfaces.

