# Supplementary material for: Phosphate-Induced Reaction to Prepare Coal-Based P-Doped Hard Carbon with a Hierarchical Porous Structure for Improved Sodium-Ion Storage
Source: Molecules. 2023 Jun 22;28(13):4921. doi: 10.3390/molecules28134921 (PMC10343881; doi:10.3390/molecules28134921)
Supplement: Supplementary file 1 [file molecules-28-04921-s001.zip › molecules-2441647-supplementary.pdf]

Supplementary information

Table S1 The proximate and ultimate analyses of subbituminous coal.

Ultimate analyses (daf $_{wt}$%)					Proximate analyses ($_{wt}$%)			
C	H	O	N	S	M_{ad}	A_d	V_{daf}	FC_d
81.24	4.47	9.29	0.79	0.46	1.48	3.76	35.08	62.48%

Ad: air dried basis; d: dry basis; daf: dry and ash-free basis.

Table S2 The ash composition analyses (wt%) of subbituminous coal.

CaO	Fe_2O_3	SO_3	SiO_2	Al_2O_3	MgO	Na_2O	MnO_2	TiO_2	K_2O	P_2O_5	etc.
23.24	20.98	17.56	17.16	7.53	6.94	0.98	0.74	0.64	0.26	0.05	3.92

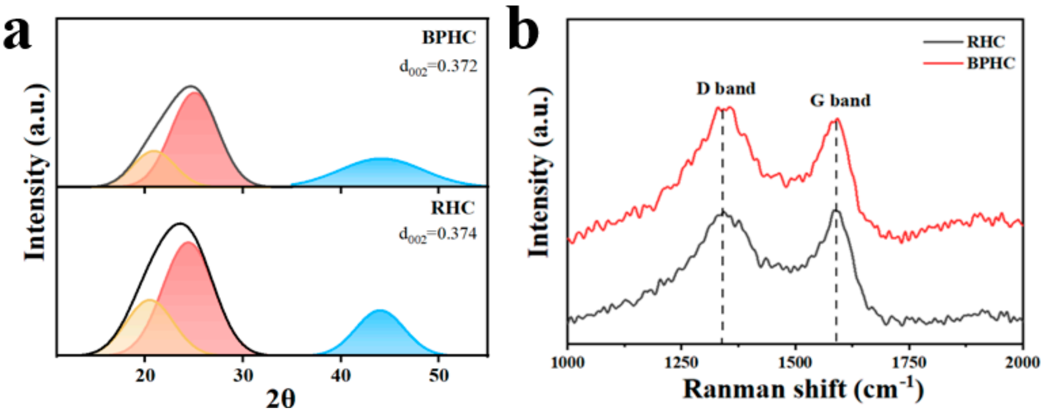

Figure S1 Fitted XRD patterns and Raman spectra of RHC and BPHC (a,b).

Table S3 The XRD (002) peak fitting analysis for RHC and BPHC.

Sample	$2\theta_{002}$ (°)	d002 (nm)	La (nm)	$2\theta_{001}$ (°)	Lc (nm)
RHC	23.95	0.371	2.02	44.19	1.51
BPHC	23.82	0.373	1.81	44.09	1.43

Table S4 Deconvoluted band assignments of Raman spectra.

**Band**	**Raman Shift(cm^{-1})**	**Corresponding structure**	**Vibration mode**	**Line Shape**
**G**	~1580	Graphitic lattice	E_{2g} Symmetry	Lorentz
**D(D1)**	~1350	Disordered graphitic lattice	A_{1g} Symmetry	Gauss+Lorentz
**D2**	~1620	Few-layer graphene	E_{2g} Symmetry	Lorentz
**D3**	~1500	Amorphous structure	--	Gauss
**D4**	~1200	sp^2-sp^3 hybrid structures or C-C/C=C stretching vibrations	A_{1g}Symmetry	Lorentz

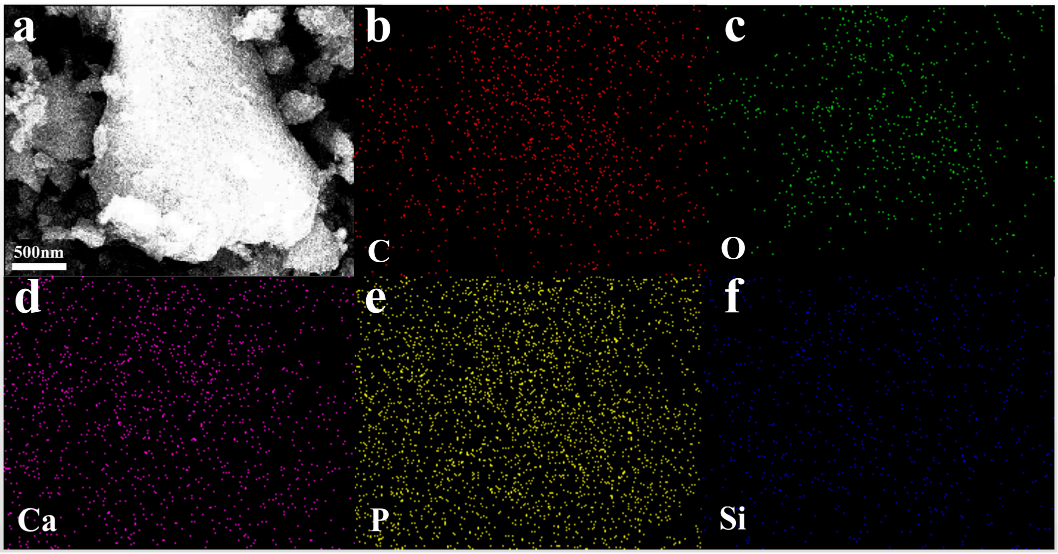

Figure S2 SEM and EDS elemental mappings of BPHC; SEM image of BPHC (a); elemental mappings for (b) C, (c) O, (d) Ca, (e) P and (f) Si of BPHC based on (a).

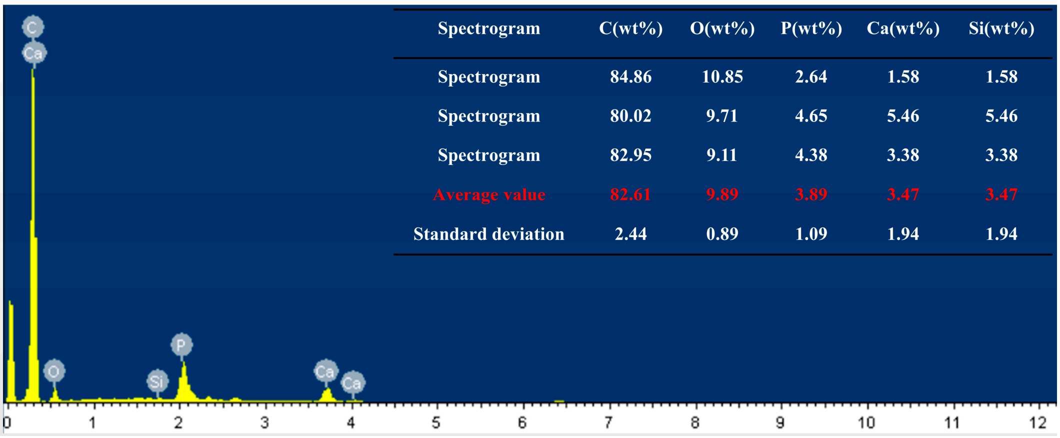

Spectrogram	C(wt%)	O(wt%)	P(wt%)	Ca(wt%)	Si(wt%)
Spectrogram	84.86	10.85	2.64	1.58	1.58
Spectrogram	80.02	9.71	4.65	5.46	5.46
Spectrogram	82.95	9.11	4.38	3.38	3.38
Average value	82.61	9.89	3.89	3.47	3.47
Standard deviation	2.44	0.89	1.09	1.94	1.94

Figure S3 EDS quantification of C, O, P, Ca and Si elements for BPHC.

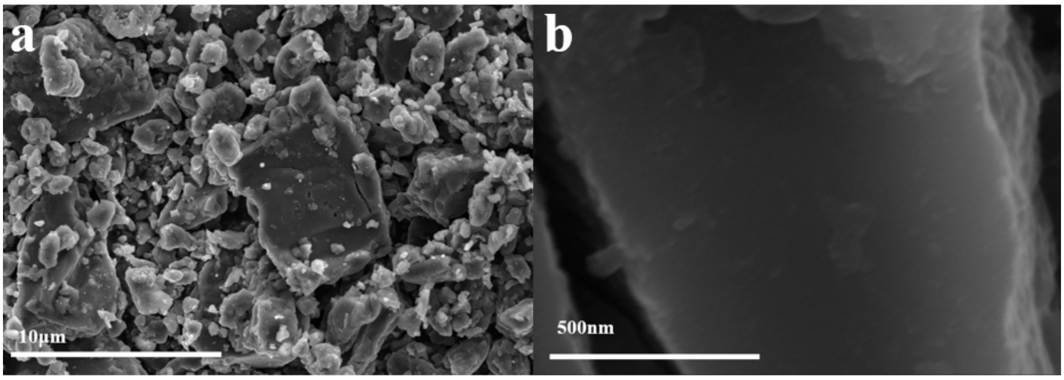

Figure S4 SEM images at different magnifications of RHC (a,b).

Table S5 Data calculated by EIS of RHC and BPHC.

Sample	σ	Rct	$D_{Na+/}$cm s^{-1}
RHC	2631.9732	712.5	$1.1090*10^{-19}$
BPHC	553.3679	140.3	$2.5087*10^{-18}$
